# Strengthening frontline capacity for skin neglected tropical diseases: Findings from a global survey

**DOI:** 10.1371/journal.pntd.0014309

**Published:** 2026-05-18

**Authors:** Sarah Anwar, Esther E. Freeman, Roos Geutjes, Christine Fenenga, Priya Pathak, Daniel A. Dagne, Albis F. Gabrielli, L. Claire Fuller, José Antonio Ruiz Postigo

**Affiliations:** 1 Department of Dermatology, Massachusetts General Hospital, Boston, Massachusetts, United States of America; 2 Tufts University School of Medicine, Boston, Massachusetts, United States of America; 3 InfoNTD/e-learning.infoNTD, Amsterdam, The Netherlands; 4 World Health Organization, Geneva, Switzerland; 5 London Bridge Hospital, London, United Kingdom; 6 International Foundation for Dermatology, London, United Kingdom; University of Bremen: Universitat Bremen, GERMANY

## Abstract

**Background:**

Skin-related neglected tropical diseases (skin NTDs) remain a major source of morbidity in resource-limited settings. These conditions include Buruli ulcer, chromoblastomycosis and other deep mycoses, cutaneous leishmaniasis, post–kala-azar dermal leishmaniasis, leprosy, lymphatic filariasis, mycetoma, noma, onchocerciasis, scabies, tungiasis, and yaws. Recent efforts have focused on developing training materials to help frontline health workers diagnose and treat skin NTDs. However, little is known about how these materials are perceived and utilized in endemic regions. We performed a global online survey to identify gaps and establish priorities for future training resource development.

**Methods:**

We conducted a cross-sectional, anonymous online survey to assess training needs for skin NTDs. The survey was disseminated by the World Health Organization’s Global Neglected Tropical Diseases Programme via the WHO website, targeted email distribution to NTD professionals, and outreach on LinkedIn. Respondents identified skin NTDs most urgently requiring improved training resources in their settings and reported on priority topics, target audiences, languages, formats, and barriers to access.

**Results:**

A total of 308 participants from 48 countries completed the survey during the one-month response period. The three most frequently reported skin NTDs in need of additional training resources were leprosy (64.6%), scabies (60.7%), and lymphatic filariasis (55.6%). Early detection and diagnosis were the most prioritized training topics (86.0%). Most respondents preferred English-language resources (62.7%), with printed manuals and guidelines identified as the most useful format (70.1%). The most commonly reported barrier was limited availability of up-to-date materials (75.3%).

**Conclusions:**

We highlight persistent gaps in the accessibility of training materials for skin NTDs. While many high-quality resources exist, their dissemination, adaptation, and translation remain limited. Respondent-identified priorities provide a framework to enhance training resources, strengthen the capacity of frontline healthcare workers, and ultimately advance global skin NTD control, elimination, and eradication goals.

## Author summary

Skin neglected tropical diseases (skin NTDs) cause significant morbidity in resource-limited settings yet often receive limited attention in global health programs. Frontline healthcare workers play a critical role in recognizing and managing these diseases, but their ability to do so depends on having access to high-quality training materials. In this study, we conducted the first global survey to understand the training needs of those caring for patients with skin NTDs. Over 300 professionals from 48 countries shared their perspectives on which diseases require the most urgent attention, which topics and formats are most useful, and the barriers they face in accessing resources. Our findings reveal a disconnect between previously documented gaps in existing resources and the real-world needs of healthcare workers, highlighting the need for improved dissemination, adaptation, and translation of currently available materials. Addressing these priorities can strengthen the capacity of frontline workers and advance global efforts to control and eliminate skin NTDs.

## Introduction

Neglected tropical diseases (NTDs) affect more than one billion people across the world and pose major public health challenges [1]. Many of these conditions have significant skin manifestations and are known as “skin NTDs” [2]. Since skin manifestations are often the earliest signs of disease, frontline healthcare workers can play a key role in early recognition and management of skin NTDs. However, healthcare workers are often limited by gaps in training and inadequate access to high-quality educational materials [3,4].

The World Health Organization (WHO) is working to identify deficiencies in current training resources and to develop and disseminate updated materials [5]. In a recent scoping review of online training materials, we identified high-quality resources for each skin NTD and noted that other existing resources frequently lacked images of skin manifestations, coverage of disease complications and follow-up care, interactive features, and availability in local and regional languages [3]. While such work has highlighted areas for improvement, it is also important to capture perspectives of those most affected by these gaps and integrate their priorities into future resources. We conducted a global needs assessment survey among providers involved in care of patients with skin NTDs. We aimed to characterize their perceived challenges in accessing and utilizing current resources, identify priority diseases and target audiences, and understand preferences for format and language to inform the development of future educational tools.

## Materials & methods

We conducted a cross-sectional, anonymous online survey to characterize perceived challenges and priorities related to training for skin NTDs. The survey was informed by findings from our prior scoping review of existing training resources for skin NTDs, which identified gaps in content and accessibility [3].

The online questionnaire was disseminated by the World Health Organization’s (WHO) Global Neglected Tropical Diseases Programme. The survey was published on the WHO website in English, Spanish, and French. It was also disseminated via targeted email to NTD professionals, including members of the WHO’s Skin Neglected Tropical Diseases Capacity Strengthening and Training Working Group and national NTD program managers. In addition, information about the survey was shared on LinkedIn by an NTD-focused non-governmental organization leader based in Tanzania, who was not part of the study team and independently raised awareness of the study among professional networks in the field. Data collection was conducted over one month from January 23, 2025, to February 23, 2025. Participation was voluntary and confidential; no personally identifying information was collected.

The survey consisted of multiple-choice questions that allowed participants to select all applicable responses. Participants could also select “other” and input free text. They were asked to report their occupation and country of residence and to indicate which skin NTDs they perceived as most urgently requiring improved training resources in their settings. They were also questioned on perceived gaps in existing resources, priority target audiences for new materials, key barriers in accessing current resources, and preferences regarding language and format.

The full survey questionnaire (S1 File) and de-identified survey response dataset (S2 File) are provided as Supporting Information.

Responses were collected using Microsoft Forms, and data were exported for analysis. Multiple-choice responses were analyzed using descriptive statistics. Free-text write-in “other” responses to multiple choice questions were reviewed qualitatively and grouped into common thematic categories or mapped into existing answer choices when appropriate.

This project was reviewed by the Mass General Brigham Institutional Review Board and was exempted from IRB approval, as it did not involve the collection of individually identifiable data.

## Results

### Respondent characteristics

A total of 308 participants from 48 countries spanning six continents completed the survey during the one-month response period. The countries with the highest number of respondents were Nigeria (79/308, 25.7%), Ghana (56/308, 18.2%), Ethiopia (18/308, 5.8%), Tanzania (17/308, 5.5%), India (14/308, 4.5%), Cote d’Ivoire (14/308, 4.5%), and Cameroon (11/308, 3.6%). Respondents included a range of professional roles, including healthcare workers (136/308, 44.2%), trainers or educators (77/308, 25.0%), NTD program managers (47/308, 15.3%), students (20/308, 6.5%), researchers (15/308, 4.9%), and others (13/308, 4.2%) (Table 1).

**Table 1:** Respondent characteristics.

	n (%)
Total respondents	308 (100%)
Countries represented (n = 48)	African Region – 22 (45.8%) Eastern Mediterranean – 6 (12.5%) European Region – 6 (12.5%) Region of the Americas – 5 (10.4%) South-East Asian – 5 (10.4%) Western Pacific – 4 (8.3%)
Role	
– Healthcare workers	136 (44.2%)
– NTD trainers/educators	77 (25.0%)
– Program managers	47 (15.3%)
– Researchers	15 (4.9%)
– Students	20 (6.5%)
– Other	13 (4.2%)*

* Respondents who selected “Other” and provided a write-in response were grouped into existing categories where appropriate. The remaining ‘Other’ category includes those whose roles did not fit clearly elsewhere.

### Training priorities identified

**Fig 1:**
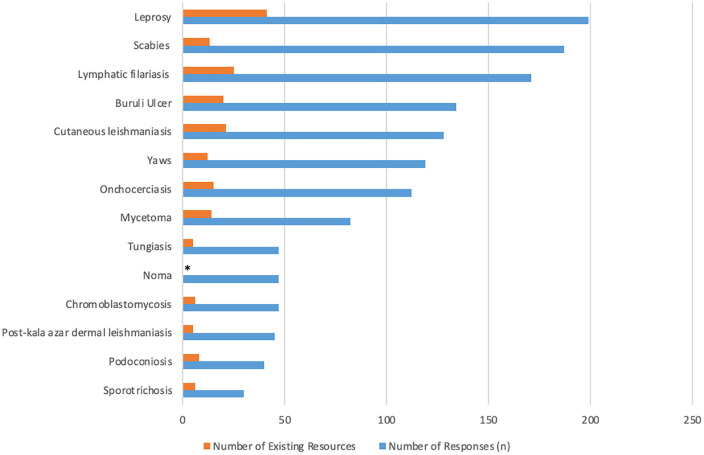
Priority Skin NTDs and Resource Availability. Number of survey respondents identifying each neglected tropical disease as a priority, compared with the number of currently available resources. *Noma was not included in the prior scoping review of online training materials [3]..

**Fig 2:**
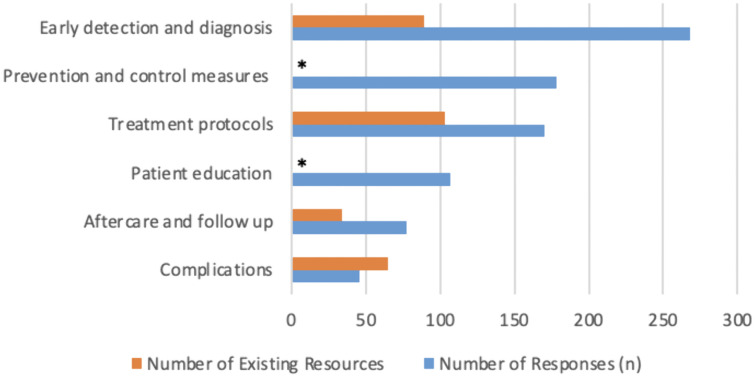
Priority Training Topics and Resource Availability. Number of survey respondents identifying each training topic as a priority, compared with the number of currently available resources. *Prevention and control measures and patient education were not included in the prior scoping review of online training materials [3].

The five most frequently reported skin NTDs urgently requiring additional training materials were leprosy (199/308, 64.6%), scabies (187/308, 60.7%), lymphatic filariasis (171/308, 55.5%), Buruli ulcer (134/308, 43.5%), and cutaneous leishmaniasis (128/308, 41.6%) (Fig 1). Early detection and diagnosis were identified as the most critical area for improvement in training resources (268/308, 87.0%), followed by prevention and control measures (178/308, 57.8%), treatment protocols (170/308, 55.2%), patient education (107/308, 34.8%), aftercare and follow-up (77/308, 25.0%), and complications (46/308, 14.9%) (Fig 2).

Respondents prioritized community health workers (274/308, 89.0%) as the key audience for new or improved training materials, followed by nurses (216/308, 70.1%), doctors (195/308, 63.3%), patients (195/308, 63.3%), and pharmacists (81/302, 26.3%). Most participants (249/308, 81%) *strongly agreed* that improving the ability to recognize and manage common skin diseases would also enhance the effectiveness of community-based NTD interventions and an additional 17% (53/308) *agreed* with this statement.

**Fig 3:**
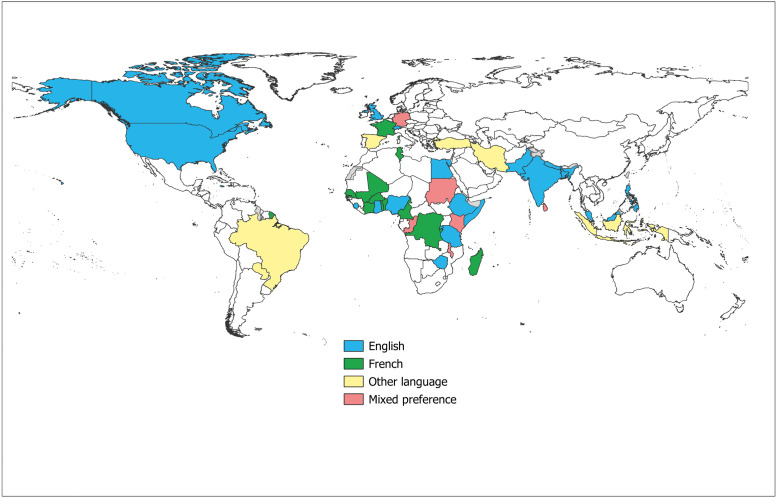
Preferred Language for Skin NTD Training Resources. Mixed preference indicates countries where respondents reported comparable preference for English or French and at least one additional language. Source: Skin NTD training resource global survey. Base Map created using Natural Earth (https://www.naturalearthdata.com/downloads/10m-cultural-vectors/10m-admin-0-countries/); terms of use: https://www.naturalearthdata.com/about/terms-of-use/.

Most respondents indicated a preference for training materials in English (193/308, 62.7%), while a smaller number preferred French (48/308, 15.6%)**,** Spanish (7/308, 2.3%)**,** Portuguese (4/308, 1.3%)**,** and Arabic (4/308, 1.3%) (Fig 3). However, 16.8% (52/308) of respondents selected “other” most commonly indicating a need for resources in local or regional languages, particularly those spoken in Africa (45/52, 86.5%). Printed manuals or guidelines (216/308, 70.1%)**,** videos (197/308, 64.0%), and interactive online courses (195/308, 63.3%) were rated as the most useful formats for training materials. Other commonly selected formats included mobile applications for field use (180/308, 54.4%)**,** webinars or live training sessions (174/308, 56.5%), audio training messages (77/308, 25.0%), and podcasts (58/308, 18.8%)**.**

The most reported barrier to using skin NTD training resources was limited availability of up-to-date materials (232/308, 75.3%). Other challenges included a lack of materials in local languages (146/308, 47.4%), limited time for training (125/308, 40.6%), and lack of internet access (99/308, 32.1%).

## Discussion

This global survey highlighted persistent challenges and training priorities for healthcare workers who care for patients with skin NTDs. By surveying individuals across 48 countries and 6 continents, we identified the diseases and training topics that are considered most urgent as well as barriers that hinder utilization of existing resources. This work bridges a critical gap between the previously documented availability of training materials and real-world demand by those who rely on these resources at the front lines.

Respondents consistently identified leprosy, scabies, lymphatic filariasis, Buruli ulcer, and cutaneous leishmaniasis as priority diseases in need of improved training resources. This aligns with case detection studies and disease burden estimates from Liberia, Cameroon, Cote d’Ivoire, and Ghana [6,7]. However, this finding also reveals a striking disconnect as our prior scoping review demonstrated that four of these five same conditions, excluding scabies, have the greatest number of existing training resources among all skin NTDs, including several high-quality examples [3]. Thus, our combined findings suggest the problem is not just a lack of training content but rather limited dissemination and translation of existing materials to ensure they reach those who need them most. These priorities may in part reflect the geographic distribution of respondents, with a substantial proportion based in West African countries where several of these diseases are endemic.

Similarly, early detection and diagnosis emerged as the highest priority training topic lacking sufficient coverage. This finding echoes other studies of delayed diagnosis and the importance of healthcare worker training in improving case detection of leprosy and scabies [8–10]. This also contrasts from our earlier review, which demonstrated that diagnosis of skin NTDs is well covered in existing materials and further suggests that awareness and accessibility of current resources are lacking [3]. Interestingly, topics such as complications and follow-up care were not prioritized by survey participants but remain underrepresented in current materials [3]. This divergence indicates that documented gaps do not always align with priorities and reinforces the need to consider user perspectives when informing future resource development.

The emphasis on community workers as a key target audience for skin NTD trainings aligns with the WHO’s NTD strategic framework and roadmap for 2021–2030 [5]. In their plan, the WHO emphasizes the importance of capacity building among this group and envisions them as key actors in integrated care [5]. Prior studies have also demonstrated that training community health workers can improve case detection of multiple skin NTDs in endemic countries [6,11,12]. At the same time, respondents indicated interest in training resources for nurses, doctors, pharmacists, and patients, indicating that multi-level training strategies may be necessary to strengthen the continuum of care.

Respondent preferences for resource language and format also provided important insights. While most respondents prioritized resources in English, the frequent demand for materials in French and local African languages reinforces an existing challenge and underscores the importance of multilingual translation to reach a broader audience [3]. Respondents valued a diversity of resource formats, including printed manuals and guidelines, videos or other visual aids, and online interactive courses. These answers indicate a wide variety of learning preferences. Notably, traditional manuals were highly valued despite recent efforts to prioritize digital and interactive tools [13]. Preferences for printed versus digital formats may also reflect varying levels of comfort with technology among survey respondents, which was not explicitly addressed in the survey. While printed materials offer accessibility in settings with limited internet connectivity, they often lack high quality images, raising concerns about their ability to familiarize trainees with disease signs and support diagnosis in practice [3]. Future training efforts should also consider cultural appropriateness and contextual relevance when adapting materials for local settings.

Lastly, the limited availability of up-to-date resources was a substantial reported barrier, while lack of translation, time constraints, and poor internet connectivity were noted as additional but less prominent issues. Similar challenges have been documented in other disease programs in resource-limited settings, including infectious diseases and maternal and newborn care [14,15]. Together, these findings reinforce the need to prioritize dissemination, updating, and translation of existing resources to ensure accessibility in countries with high NTD burdens. This study offers a basis for strengthening training on skin NTDs among frontline healthcare workers worldwide. However, several limitations should be considered. First, this was a cross-sectional self-reported survey, so findings represent respondent perspectives at a single point in time and may have been influenced by selection bias. Although healthcare workers were the largest group represented, the survey did not collect cadre-specific details (e.g., community health workers, nurses), limiting our ability to examine how training priorities may differ across the diverse roles encompassed by the term “healthcare worker” and to assess whether specific groups may have been over- or underrepresented. This gap could have influenced training topics prioritized if certain groups were more heavily represented than others. Further, the survey was distributed through WHO networks and online platforms. As a result, respondents were likely already engaged in formal NTD programs, and healthcare providers outside these networks may have been missed. In addition, the extent to which providers of informal or traditional healthcare participated in the survey is unclear, despite their important role as first points of care for skin NTDs in many settings. Lastly, while the survey was available in English, French, and Spanish, language accessibility and dissemination channels may have limited participation in certain regions. For example, there appeared to be limited reach to Spanish-speaking countries, suggesting the survey may not have captured perspectives from all global regions equally. In addition, the high preference reported for English and French resources could partially reflect the survey languages and networks through which the survey was distributed. Perspectives from providers from other language regions may be underrepresented. These factors should be considered when interpreting the results, as they may affect the generalizability of the findings despite the wide geographic diversity of respondents.

In conclusion, we conducted the first global survey to identify priorities for training materials on skin NTDs. Our findings underscore a persistent gap between resource availability and accessibility, emphasizing the need to complement resource development with strategies for adaptation and dissemination to maximize their impact and reach. Future efforts should address linguistic and technological barriers and provide multi-audience training to strengthen the capacity of healthcare workers and advance global control and elimination goals for skin NTDs.

## Supporting information

S1 File Survey questionnaire. Full questionnaire used in the global survey on skin NTD training needs.
(PDF)

S2 File Survey response dataset. De-identified dataset containing responses from the global survey on skin NTD training needs, including multiple-choice and write-in responses. Data are provided as an Excel (.xlsx) file.
(XLSX)
