# Supplementary material for: Strengthening frontline capacity for skin neglected tropical diseases: Findings from a global survey
Source: PLoS Negl Trop Dis. 2026 May 18;20(5):e0014309. doi: 10.1371/journal.pntd.0014309 (PMC13211258; doi:10.1371/journal.pntd.0014309)
Supplement: S1 File — Full questionnaire used in the global survey on skin NTD training needs. (PDF) [file pntd.0014309.s001.pdf]

Needs Assessment Survey for Training in Skin NTDs

The WHO Global Neglected Tropical Diseases Programme is conducting a brief survey to better understand the training needs of frontline healthcare workers in caring for patients with skin-related neglected tropical diseases (skin NTDs). We have been working to prepare training materials, and we aim to identify key priorities to guide future resource development.

We kindly ask you to take a few minutes to share your insights through this survey. Thank you for your time and participation! The survey is available in English, Spanish and French.

Please note that your survey answers will be stored and analysed confidentially. We appreciate your participation and look forward to receiving your feedback.

Yours sincerely,

The Skin NTD Team and The Knowledge Management Team
Global Neglected Tropical Diseases Programme
World Health Organization

Survey Questions:

1. **What is your profession?**
   - Healthcare worker
   - Trainer/educator
   - Program manager
   - Student
   - Other

2. **Where do you currently live?** (Drop-down menu)

3. **Which skin neglected tropical diseases (NTDs) most urgently require additional or improved training materials for healthcare workers in your region??**
   (Select all that apply)
   - Buruli ulcer
   - Chromoblastomycosis
   - Cutaneous leishmaniasis
   - Leprosy
   - Lymphatic filariasis
   - Mycetoma
   - Noma
   - Onchocerciasis (river blindness)
   - Podoconiosis

- Post-kala-azar dermal leishmaniasis
- Scabies
- Sporotrichosis
- Tungiasis
- Yaws

4. **Which areas of training materials for NTDs do you think need the most improvement?**
   (Select up to 3)
   - Early detection and diagnosis
   - Treatment protocols
   - Complications
   - Aftercare and follow up
   - Prevention and control measures
   - Patient education

5. **Which priority audiences should these resources be targeted towards?** (Select all that apply)
   - Nurses
   - Doctors
   - Pharmacists
   - Community Health Workers
   - Patients

6. **To what extent do you agree with the following statement?** Improving the ability to recognize and manage common skin diseases can support integrated approaches for community-based NTD interventions, given that many NTDs have skin manifestations that can aid in early diagnosis."
   - Strongly agree
   - Agree
   - Neutral
   - Disagree
   - Strongly disagree

7. **What are the biggest barriers you face when accessing or using NTD training materials?**
   - Lack of internet access
   - Materials not in the local language

- Limited availability of updated resources
- Lack of time for training
- Other

8. **What is your preferred language for training materials?**
    - Arabic
    - Chinese
    - English
    - French
    - Portuguese
    - Russian
    - Spanish
    - Other

9. **What types of resources would be most helpful for training healthcare workers on NTDs?**
   (Select all that apply)
    - Interactive online courses
    - Printed manuals or guidelines
    - Webinars or live training sessions
    - Mobile apps for field use
    - Videos or other visual aids
    - Audio training messages
    - Podcasts
    - Other

10. **Do you have any additional feedback or suggestions regarding NTD training materials?** (Free text)
